# Spontaneous emergence of context-dependent statistical learning in humans and neural networks

**DOI:** 10.1016/j.isci.2026.116640

**Published:** 2026-08-12

**Authors:** Fleming C. Peck, Hongjing Lu, Jesse Rissman

**Affiliations:** 1 Department of Psychology, University of California, Los Angeles, Los Angeles, CA, USA; 2 Department of Statistics, University of California, Los Angeles, Los Angeles, CA, USA; 3 Department of Psychiatry and Biobehavioral Sciences, University of California, Los Angeles, Los Angeles, CA, USA

**Keywords:** statistical learning, context, recurrent neural networks, predictive coding

## Abstract

Humans readily extract statistical regularities from experience, yet natural environments require flexible adaptation when associative structures shift across changing contexts, often without warning. Across two experiments, we show that humans can incidentally learn overlapping and conflicting visual associations even when contexts dynamically alternate and remain unsignaled or only minimally cued. To probe the computational mechanisms supporting this adaptive capacity, we trained recurrent neural networks with gated recurrent units on the same statistical learning task without providing any explicit context information. Models with moderate initialized weight variance demonstrated context-dependent learning and most closely matched human learning behavior. These models spontaneously developed distributed internal representations of context that robustly separated conflicting associations and supported rapid adaptation to latent context shifts. Together, these behavioral and computational results advance our understanding of how humans and artificial systems can successfully learn and flexibly retrieve context-dependent associations under challenging conditions.

## Highlights

• Humans can incidentally learn conflicting context-dependent visual temporal associations
• Both humans and neural networks can achieve this without explicit context cues
• Optimal variance of network’s initial weights enables learning of latent structure
• Successful models develop highly distributed representations of context

## Graphical abstract

**Figure:**
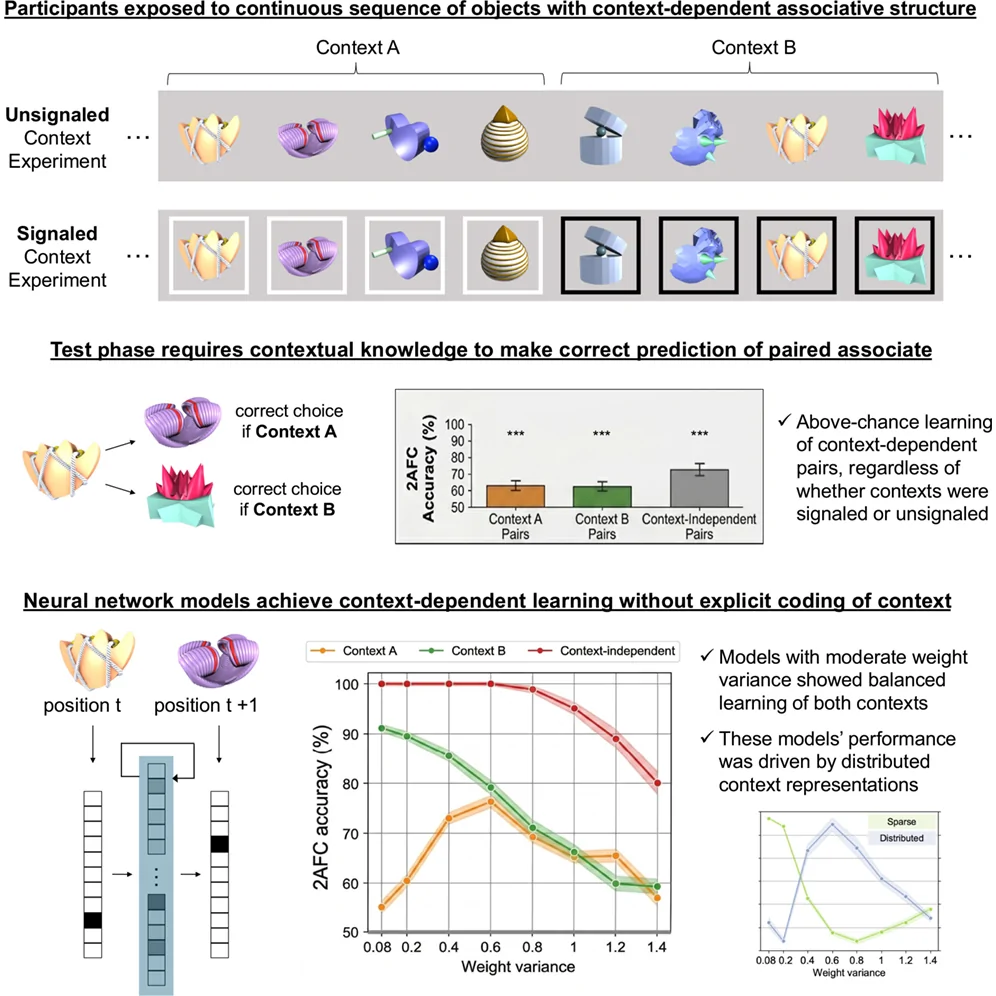

## Teaser

biological sciences; psychology; cognitive neuroscience; computational bioinformatics; neural networks

## Introduction

Many everyday experiences unfold in structured, predictable ways, with events that recur over time in stable patterns. Internalizing these regularities allows anticipation of future occurrences, facilitating efficient information gathering, decision-making, and behavioral adaptation. It follows that the human brain is fundamentally oriented toward predicting upcoming events based on recent experience.^1^^,^^2^^,^^3^ This predictive ability helps conserve cognitive resources by reducing the need for continuous, effortful learning once patterns have been identified.^4^ However, the world is rarely static: associations often vary across contexts.^5^ To support adaptive behavior, the brain is thought to engage in context-dependent learning of these regularities and associations for flexible predictions as environmental conditions shift.^6^ For example, navigating a daily commute relies on learning the timing and location of traffic congestion, and expectations for social interaction may differ when a friend is encountered at work versus at a party. In both cases, prior experience supports the formation of context-bound predictions that guide perception and behavior.

Humans have an innate ability for statistical learning, allowing them to spontaneously discover regularities and associations. This process extracts spatial and temporal regularities from sensory input through passive exposure, without explicit instruction or external rewards.^7^ Statistical learning is proposed to support a wide range of cognitive functions, including language acquisition, visual perception, object recognition, and social cognition.^8^^,^^9^ Empirical studies demonstrate that individuals can detect regular patterns in continuous streams of stimuli across visual,^10^ auditory,^11^ and tactile^12^ modalities, in the absence of explicit transition cues and instructions.

Despite the rich literature on statistical learning, most research has focused on simple, highly reliable associations, such as detecting short sequences of objects or sounds. However, in natural environments, context plays a critical and often unobserved role in shaping how associations are formed. In animal learning research, for example, association-based behavior is known to be highly context-specific: extinguished fear responses return when animals are tested outside the extinction setting.^13^ Moreover, following extinction or reversal learning, animals reacquire original contingencies more rapidly than during initial learning,^14^^,^^15^ suggesting that prior contingencies are retained as latent knowledge in memory rather than being overwritten by new learning. Cognitive control processes are thought to underpin the behavioral flexibility afforded by suppressing previously useful but no longer relevant responses, allowing learners to pivot between contexts and contingencies as the environment demands.^16^ Notably, most studies in the animal learning literature involve explicit reinforcement (e.g., reward or punishment), whereas statistical learning occurs incidentally without feedback, instruction, or overt motivation.

Although a few studies have explored the statistical learning of regularities that depend on a latent context or environment (e.g.,^17^^,^^18^^,^^19^^,^^20^^,^^21^), it remains unclear whether individuals can incidentally learn and retrieve context-dependent temporal associations without explicit perceptual context cues, reinforcement, or instruction. Analogous mechanisms have been proposed in sensorimotor learning, an instance of implicit learning where the brain is thought to infer context shifts and partition experience into distinct memories.^22^ Here, we test whether people can acquire two distinct sets of temporal associations instantiated with an overlapping pool of visual objects, where most associations are in direct conflict between contexts. For example, in Context A, object X is followed by object Y, whereas in Context B, the same object X is followed by object Z. Successful learning requires participants to flexibly update their expectations according to the active context inferred from recent sequence history. We examine how well human learners can discover these context-dependent associations without any external context cue—where context is embedded only in the pattern of transitions—using both offline testing and online learning measures.

To explore how these context-dependent representations might emerge from experience, we trained neural network models on the same behavioral task. We then identified the model that best matched human performance across the experimental conditions and analyzed its hidden-layer activations to generate testable hypotheses about analogous representations in the human brain. Deep neural networks have proven effective at capturing lower-level sensory processing,^23^ and recent perspectives advocate for extending these approaches to the study of higher-order cognition, including the representation of abstract knowledge.^24^ However, a common limitation of these modeling efforts is that these networks are typically trained on far more data than human learners,^25^ limiting the validity of direct comparisons. Additionally, prior modeling work often incorporates strong inductive biases that render context artificially explicit, either by providing an unambiguous context signal as part of the input^21^^,^^26^ or by augmenting network architecture with designated units or computation modules for encoding context.^27^^,^^28^ These modifications, while effective, constrain opportunities to observe how context discovery might emerge spontaneously. Inspired by Elman’s finding that simple recurrent neural networks can capture both short- and long-range dependencies^29^ and echoing recent calls to avoid hard-wiring solutions in cognitive modeling,^30^ we used minimally structured architectures that omitted context signaling and specialized inference modules. This design allowed us to examine how networks discover and represent latent task structure through sequence exposure alone. Finally, given evidence that weight initialization scale can influence learning trajectories in neural networks,^26^^,^^31^^,^^32^ we systematically varied the initial weight magnitudes of the networks to assess how this factor affects their ability to learn and distinguish context-dependent associations.

The goal of the neural network modeling is to generate hypotheses about how the brain might represent context in statistical learning. The hippocampus utilizes two dominant neural representation strategies to support memory of individual experiences and to extract regularities across experiences:^33^^,^^34^ sparse and distributed coding. Sparse codes, observed in the dentate gyrus and CA2/3 subregions, involve highly selective activation of a small subset of units in response to a given input.^35^ Distributed representations, observed in the CA1 subregion, encode inputs across overlapping patterns of activity spanning the neural population.^36^ Here, we use this distinction as a conceptual framework to characterize how contextual information is represented within the model’s hidden layer. We quantify the extent to which context sensitivity is concentrated in a small subset of hidden layer units versus distributed across units, and we relate these representational properties to task performance.

Overall, this study aims to advance our understanding of context-dependent statistical learning by examining whether humans can learn and retrieve multiple conflicting statistical structures within highly overlapping stimulus sets. By manipulating the presence of visual contextual cues, we assess whether explicit signals of context shifts facilitate learning and whether individuals can still learn context-dependent associations in their absence. In parallel, we use neural network models trained on the same task to test whether artificial systems can account for human-like learning dynamics, offering insight into the computational mechanisms that may support flexible, context-sensitive learning in the brain.

## Results

**Figure 1:**
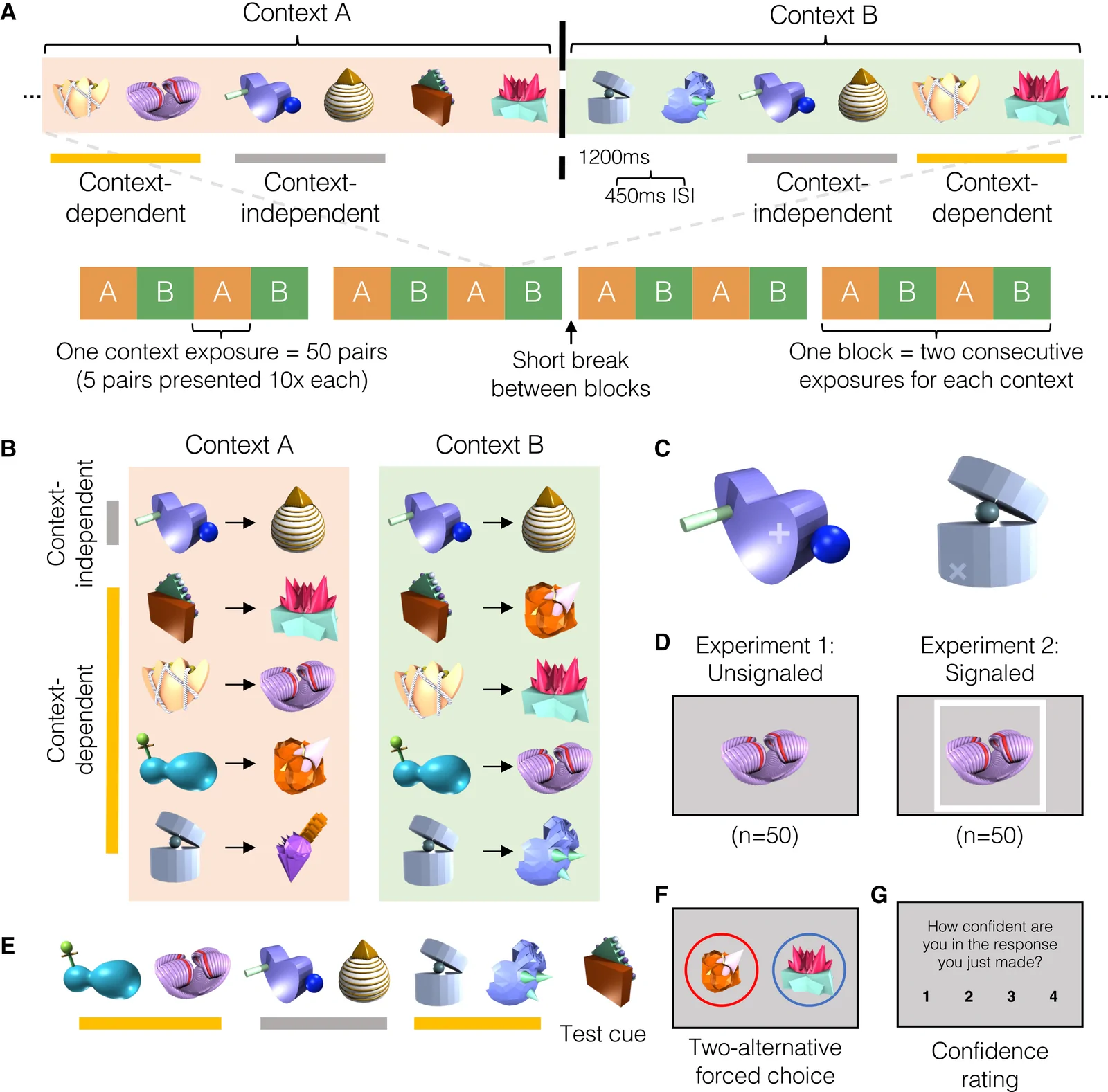
Experimental overview (A) Visualization of the learning phase. Participants viewed a uniformly paced sequence of objects separated by brief fixation periods. Each object appeared for 1200 ms with a 450 ms interstimulus interval. The sequence was organized according to the temporal pair structure dictated by one of two contexts (Context A and Context B), which switched every 50 pairs. The orange and green backdrops are shown for illustrative purposes only. Participants performed four blocks of 200 pairs each, separated by short breaks. (B) Sample object assignments to context pair structures comprising 11 unique objects. The context-independent pair is the same for both contexts as shown in the first row, three of the context-dependent pairs consist of the same object set with pair assignment of the second pair position different for each context as shown in rows 2–4, and one context-dependent pair consists of a context-specific object in the second pair position as shown in the last row. (C) Example of objects embedded with “+” or “×”. Participants were tasked with making a button-press response to indicate which symbol each object contained; object-symbol mapping was held constant throughout the experiment. (D) Differentiation of the two experiments: in Expt. 1 (unsignaled), no context cues were shown and thus context switches were entirely latent (left); in Expt. 2 (signaled), context was indicated with a white or black border around the object (right). (E) 2AFC test procedure: example of 6-item (3-pair) sequence leading up to the test cue of a 2AFC trial. (F) Immediately following the test cue, participants chose which of two candidate objects comes next in the sequence. This example is a direct-conflict context-dependent trial, in which the lure corresponds to the object paired with the test cue in the other context. (G) After each choice, participants made a confidence rating.

Participants performed a context-dependent statistical learning task in which they viewed a continuous stream of 1600 object images (Figure 1A). Their only task was to indicate whether a “×” or “+” was embedded on each object (Figure 1C), a perceptual judgment designed to maintain attention and allow tracking of online learning via reaction times (RTs). Unbeknownst to participants, the image stream was structured into object pairs specific to one of two distinct contexts. Although they were told that parts of the sequence might become familiar over time, they received no information about the underlying structure or the existence of multiple contexts. Each context defined a unique set of temporal associations between a largely overlapping object set, such that the probability of one object following another depended on the active context (Figure 1B).

Following the learning phase, participants completed a two-alternative forced choice (2AFC) test. Because objects appeared in both contexts, the correct association on a given trial depends on the active context. Accordingly, each test trial began with a six-object sequence composed of three object pairs from a single, consistent context, followed by the first item of a test pair (Figure 1E). Participants were then tasked with choosing which of two objects should come next (Figure 1F). Context-independent trials assessed knowledge of the context-independent pair. Context-dependent trials consisted of two types: in direct-conflict trials, the lure was the object paired with the test cue in the other context; in indirect-conflict trials, the lure was an object not paired with the test cue in either context. After each choice, participants rated their confidence on a 1–4 scale (Figure 1G).

In Expt. 1 (unsignaled), *n* = 50 participants completed the task without any explicit perceptual context cue. In Expt. 2 (signaled), a separate group of *n* = 50 participants completed the same task but with a visual context cue: a colored border (white or black) surrounding each object, corresponding with active context (Figure 1D); this border was present during both the learning phase and the 2AFC test. Accuracy across the learning phase for the perceptual task was 92.2% for Expt. 1 and 92.7% for Expt. 2, indicating that participants attended to the stimuli during learning.

### Behavioral evidence of context-dependent statistical learning

**Figure 2:**
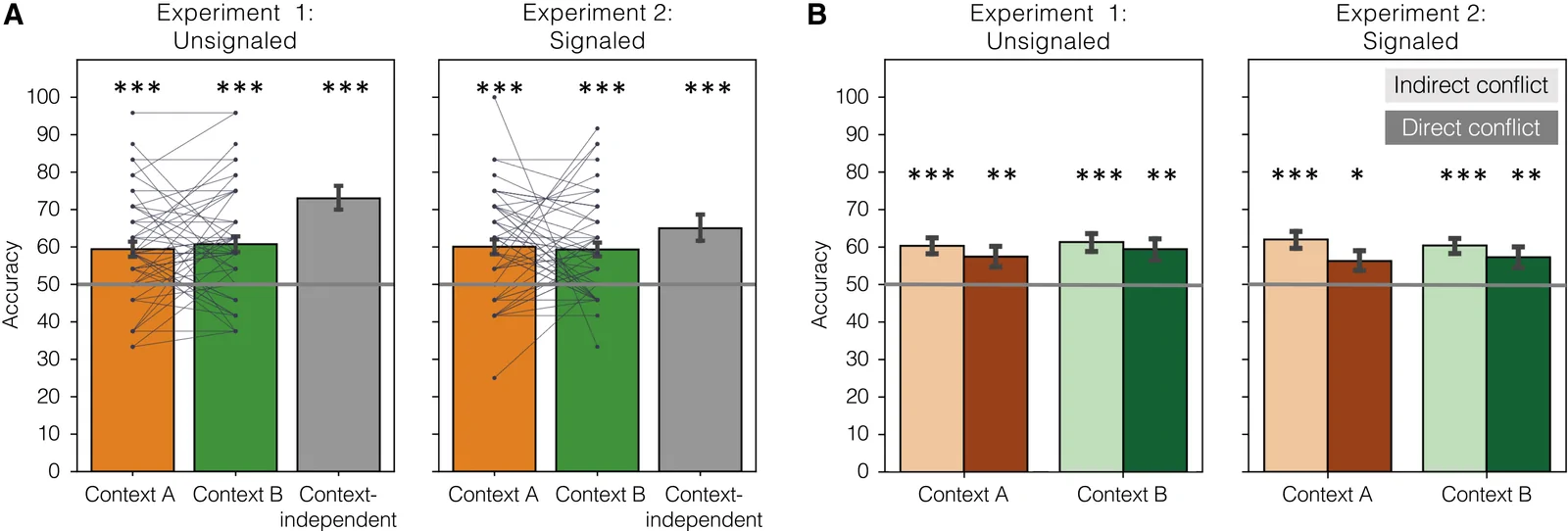
2AFC test performance Bar height reflects group average 2AFC accuracy (% correct) ± SEM. Results plotted separately for unsignaled and signaled context experiments. Asterisks indicate significant deviation from chance performance (50%; horizontal line). (A) Performance on all Context A questions (left bar, orange), Context B questions (middle bar, green), and context-independent questions (right bar, gray). Note that Context A and Context B correspond to the first and second contexts, respectively, used during the learning phase. Each dot reflects the accuracy for one participant with lines connecting a participant’s performance across the two contexts. (B) Performance on context-dependent questions, split by indirect-conflict (light coloring) and direct-conflict (dark coloring) for Context A (left, orange bars) and Context B (right, green bars). ∗∗∗*p* < 0.001, ∗∗*p* < 0.01, ∗*p* < 0.05.

We observed evidence of context-dependent statistical learning with significant 2AFC performance for both contexts (one-sample *t* tests, Holm-Bonferroni corrected for three tests, all *p* < 0.001; Figure 2A). When considering direct- and indirect-conflict context-dependent test trials separately, we found above-chance accuracy for both trial types (all one-sample *t* tests *p* < 0.05; Figure 2B). Separate mixed-design ANOVAs restricted to direct- and indirect-conflict trials with context as a within-subject factor and experiment as a between-subjects factor revealed no significant main effects or interaction effects (all *p* > 0.05). Next, a mixed-design ANOVA was conducted to examine the effects of experiment (unsignaled vs. signaled context, between-subjects) and context-dependence (context-independent vs. context-dependent trials, within-subjects) on 2AFC accuracy. There was a significant main effect of context-dependence (*F*[1, 98] = 17.52, *p* < 0.001), reflecting higher performance on context-independent than context-dependent trials. The main effect of experiment was not significant (*F*[1, 98] = 1.93, *p* = 0.17), nor was the interaction between experiment and context-dependence (*F*[1, 98] = 3.07, *p* = 0.08). We used Bayesian estimation to assess equivalence of context-dependent 2AFC accuracy between experiments. The posterior distribution of the mean difference was centered near zero (mean = 0.38%, 95% HDI [–4.1, 4.6]). Approximately 96.5% of the posterior mass fell within the predefined region of practical equivalence (ROPE) of (–5%, 5%), providing evidence that the two experiments yielded equivalent performance. This equivalence suggests that the border cue may have been too subtle to boost context-dependent learning or that explicit contextual cues are unnecessary to foster context-dependent learning beyond the contextual information that can be ascertained from recent sequence history in this paradigm. Additional analyses of confidence ratings and performance on remaining test tasks are reported in Data S1 and Figure S2. These analyses reveal that most participants showed no explicit awareness of the temporal pair structure.

We also found evidence of an online learning effect using participants’ reaction times during the learning phase when they judged whether each object contained a “×” or “+” (Figure 1C). Because these markers were consistently associated within corresponding objects across learning, faster responses could reflect memory-based predictions about the identity of upcoming objects consistent with rapid adaptation to temporal statistics in the sequence. We expect that over the course of learning, knowledge of the temporal pair structure would facilitate faster, anticipatory responses to the second item of each pair than the first item of each pair, which follows a random transition between pairs. Based on evidence that RTs improve throughout an experiment,^37^ we measure online learning as the second item RT subtracted from the first item RT, where a positive value indicates an anticipation effect, and a negative value reflects possible interference from context switches. Mean reaction times for each pair position are reported in Table S1.

**Figure 3:**
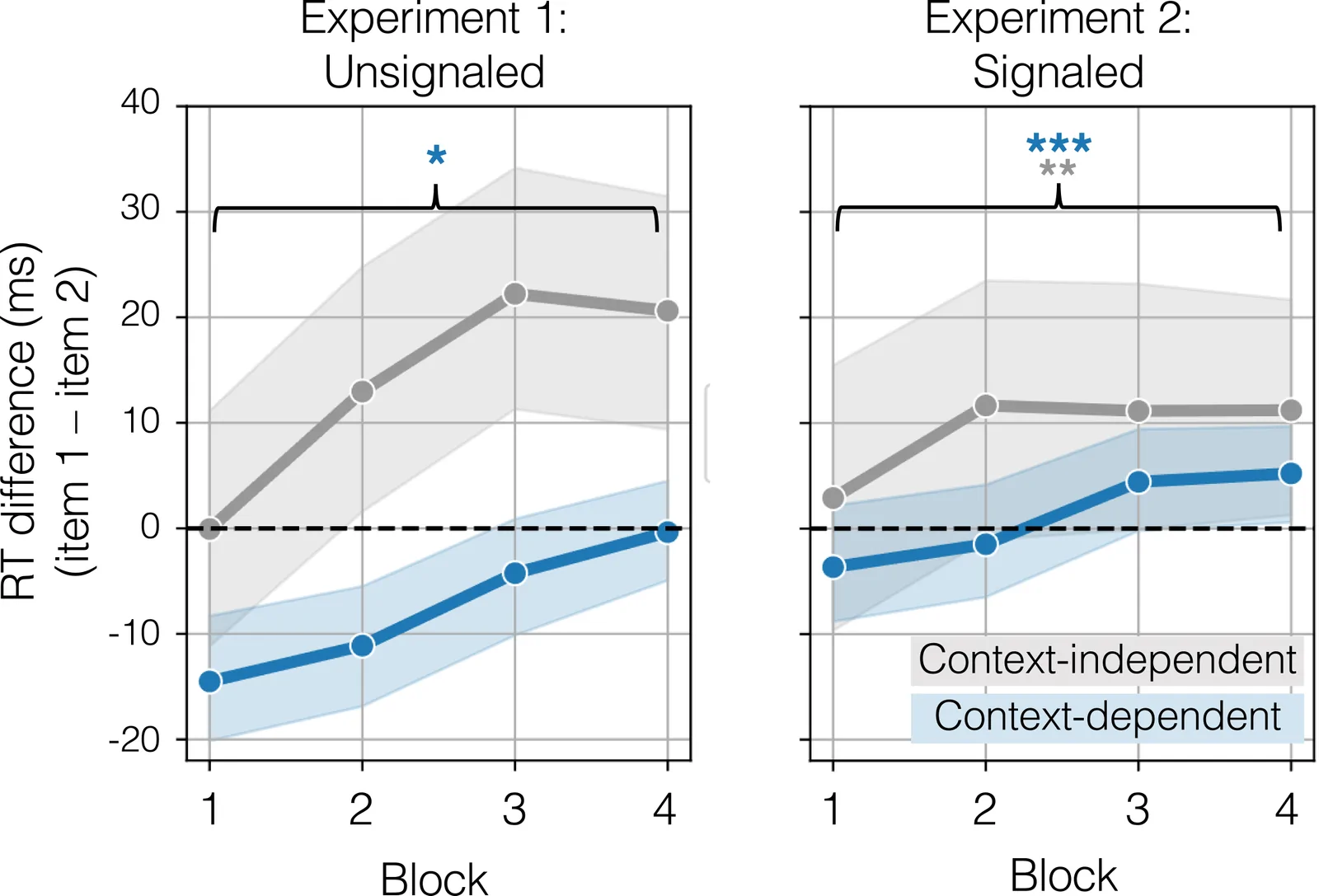
Reaction time differences reveal trajectory of online learning Reaction time (RT) difference between responses to objects in the first (item 1) and second (item 2) pair position. A positive value on the *y* axis shows anticipation effect plotted for each block during learning phase (*x* axis). Average RT difference with standard error of the mean (shaded) for context-independent pairs in gray and for context-dependent pairs in blue. Linear trend significance indicated with same color scheme. ∗∗∗*p* < 0.001, ∗∗*p* < 0.01, ∗*p* < 0.05.

For context-independent pairs (Figure 3; gray), we found a significant positive linear trend of RT differences across blocks in the unsignaled experiment (*t*[49] = 2.70, *p* = 0.009), suggesting increasing anticipatory learning over time. However, no such trend was observed in the signaled experiment (*t*[49] = 0.70, *p* = 0.49), where RT differences appeared to stabilize after the first block. For context-dependent pairs, both experiments showed a significant linear increase in RT difference across blocks (unsignaled: *t*[49] = 4.36, *p* < 0.001; signaled: *t*[49] = 2.59, *p* = 0.013). However, unlike the context-independent pairs, RTs for the predictable, item 2 objects in the context-dependent pairs of the unsignaled experiment were initially slower than the first, unpredictable items (negative RT effect) and approached equivalence by the final block. This slowing earlier in the experiment may reflect interference from frequent context switches: participants had to suppress the prediction associated with the previously active context, which would be especially demanding during the early blocks of training. This effect is slightly ameliorated in the signaled experiment, suggesting that participants may have been able to integrate the border contextual cue to facilitate online context-dependent learning. Despite this initial disadvantage for second item responses, the online learning measure increased over time, reaching its highest average in the final block. A mixed-design ANOVA on the RT difference score with experiment as a between-subjects factor and block as a within-subjects factor showed no main effect of experiment (*F*[1, 98] = 1.48, *p* = 0.23) but a significant main effect of block (*F*[3, 294] = 12.06, *p* < 0.001).

### Neural network weight initialization influences context-dependent learning

Having established that humans can spontaneously learn context-dependent associations from exposure alone, we next turned to a computational account of this behavior using artificial neural network models. Our goals were to test whether these models could similarly discover the task’s latent structure without context cues and, critically, to characterize the nature of the emergent representations that give rise to the context-dependent gating of associative predictions, examining how specific model parameters shape this capacity.

**Figure 4:**
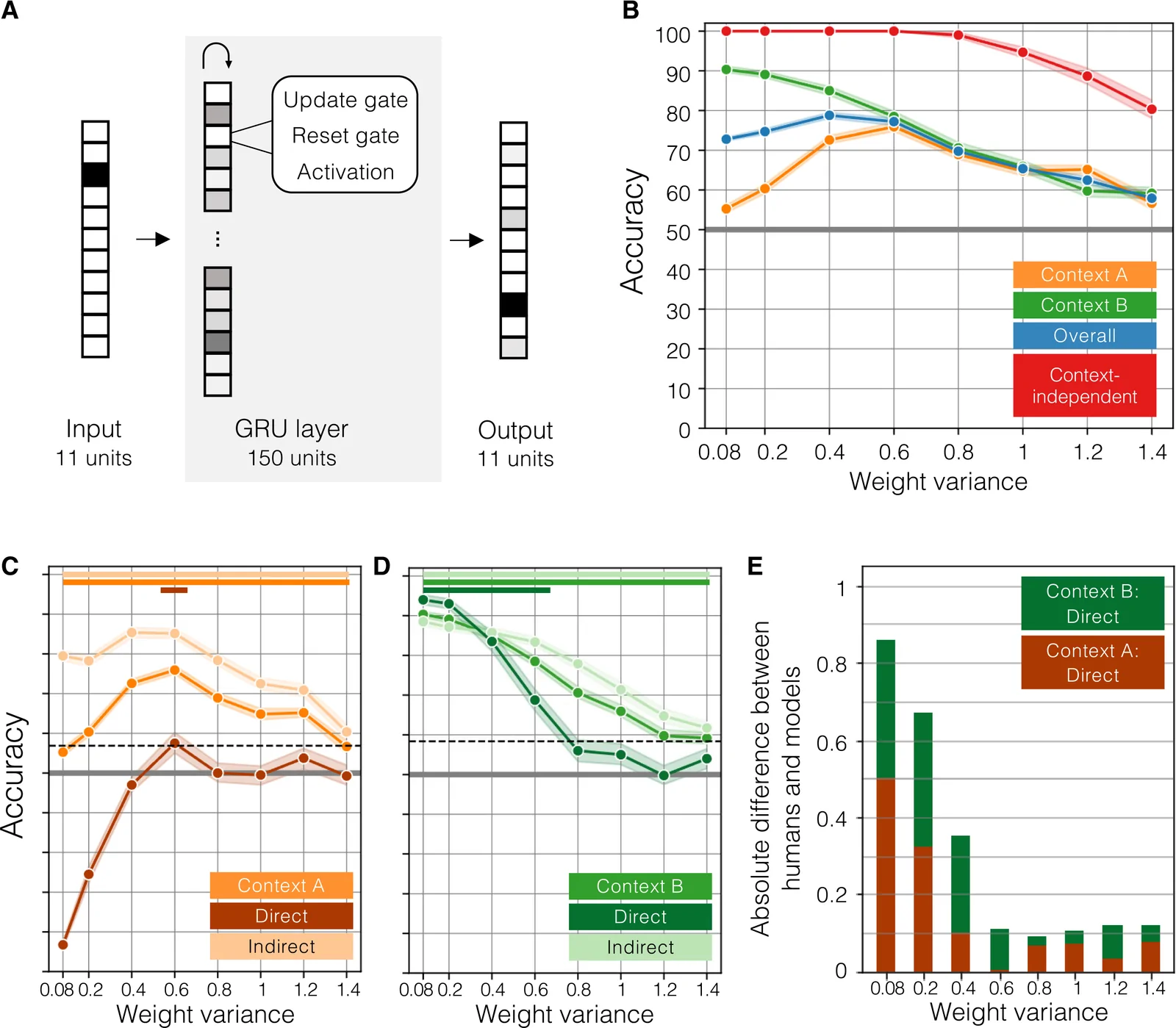
GRU model’s 2AFC performance by weight variance Data are represented as mean ± SEM. (A) Visualization of the neural network architecture, composed of 11 input units, a single GRU layer with 150 units, and 11 output units. (B–D) 2AFC accuracy (*y* axis) on context-dependent test trials for GRU models with weights initialized with increasing variance along the *x* axis color-coded by question category. (B) 2AFC performance on Context A (orange), Context B (green), overall context-dependent (blue) and context-independent (red). Chance performance (50%) indicated with gray horizontal line. (C and D) 2AFC performance on individual contexts, visualized for overall as well as direct-conflict (dark coloring) and indirect-conflict (light coloring) trial subsets. Significant one-sample *t* tests from chance (Bonferroni-corrected for eight comparisons) indicated with horizontal lines at top of plot color-coded in the same way. Mean human performance on direct-conflict trials of each context indicated by the dashed horizontal black line. (C) Context A. (D) Context B. (E) Absolute difference in direct-conflict 2AFC performance between human group average and model group average for each weight initialization configuration. Bar height reflects summed absolute difference for Context A (dark orange) and Context B (dark green).

First, we determined that recurrent neural networks with gated recurrent units (GRUs) learned the task more effectively than other network architectures, including feedforward networks and recurrent networks without gated units (see model comparison details in Methods S1 and Figure S3). Next, we trained GRU models on the same amount of sequence exposure as human participants. Models featured a 150-node hidden layer and were trained to predict the next item in the sequence using one-hot encoded object representations for both inputs and outputs (Figure 4A). Critically, models received no explicit context information, requiring them to discover the latent structure from sequence statistics to make accurate predictions. As with humans, learning was evaluated with a 2AFC test. Model weights were frozen after training, and each 2AFC trial presented the model with a series of seven objects (i.e., a three-pair sequence and a test cue). The model then “selected” the next object in the sequence between two options, with its choice determined by the object with the higher predicted probability.

We systematically varied the bounds of the uniform distribution used to initialize model weights. The manipulation is motivated by prior findings that initialization in neural networks can strongly influence learning dynamics.^38^^,^^39^ Low-variance initialization is commonly used as the default in neural networks. However, it remains unclear whether this default choice affects a model’s capacity to learn latent structures in the data, since greater initial weight variance could accelerate learning. To address this, we systematically varied the weight initialization variance across a wide range of values. For each weight variance initialization condition, we trained and tested 50 independent models and report the average performance.

Across weight initialization conditions, models with low to moderate initialized weight variance achieve perfect accuracy on context-independent trials, demonstrating their ability to learn stable, non-contextual associations (Figure 4B). However, as variance of initial weights increases, performance on context-independent trials steadily declines, highlighting how excessive initial weight variance introduces noise, disrupting the model’s ability to extract consistent patterns from the sequence.

The models’ learning of context-dependent associations—where context-specific conflicts must be resolved—reveals more complex dynamics. The relationship between initialized weight variance and context-dependent accuracy is non-monotonic, showing the highest performance by models with moderate weight initialization variance within the range of 0.4–0.6 (Figure 4B). Low-variance models (0.08–0.2) demonstrate around 90% accuracy on Context B, the context which the model was most recently processing at the end of training before model weights were frozen, compared to below 60% accuracy for Context A, the context which was previously learned and conflicted with the more recently learned associations. Increasing initialized weight variance in the high-variance range (1.0–1.4) exhibits a steady decline in accuracy for both contexts, indicating that their representations may be too diffuse or unstable, preventing them from effectively differentiating between contexts. Notably, this non-monotonic relationship between 2AFC accuracy and initialized weight variance was preserved when models were trained using input representations that reflected the perceptual features of the objects (as opposed to one-hot vectors), derived from computer vision models such as AlexNet (Methods S2 and Figure S4). The same pattern held when the context-dependent pair that included a context-unique second item was excluded, such that all pairs comprised items that appeared in both contexts (Methods S3 and Figure S5). This result helps rule out the possibility that recent exposure to particular items served as a context cue, strengthening the interpretation that context is inferred from recent sequence history.

Breaking down context-dependent performance into direct-conflict and indirect-conflict trials reveals notable differences in model learning. Direct-conflict trials (where the lure object is the correct answer for the other context) are the most diagnostic test of context-dependent learning as they place associations from different contexts in direct competition, making accurate performance dependent on the use of contextual information to disambiguate the correct response. Only models initialized with a weight variance of 0.6 achieved above-chance performance on Context A direct-conflict trials (*t*[49] = 2.81, *p* = 0.004; Figure 4C). These models also showed significant performance on Context B direct-conflict trials (*t*[49] = 6.94, *p* < 0.001; Figure 4D) and were the only models to exhibit above-chance performance on direct-conflict trials of both contexts. Low-weight models perform near floor on Context A direct-conflict trials, rendering their high overall context-dependent accuracy misleading as it reflects only strong performance on indirect-conflict Context A questions and mastery of Context B. In contrast, high-weight models show no advantage for Context B direct-conflict trials, with performance on direct-conflict questions around chance for both contexts.

To identify which weight initialization variance best approximated human-like behavior, model performance on Context A and Context B direct-conflict trials was compared to human data. Human accuracy was averaged across the unsignaled and signaled experiments as no significant difference was observed between them. Figure 4E visualizes the absolute difference in 2AFC performance between models and humans on direct-conflict trials for both contexts (human performance indicated by the dashed black line in Figures 4C and 4D). Initialized weight variance of 0.6 produced the clearest match to human performance, forming an elbow in the plot and uniquely achieving above-chance accuracy across all question sets among the tested weight configurations.

### Distributed hidden layer representation strategy facilitates context-dependent learning

Having observed that models with initialized weight variance in the moderate range (such as 0.6) successfully learned context-dependent associations without any explicit context signal as input, we next examined how context information is encoded in the hidden layer activations of the models. Context encoding could manifest as either a sparse representation (carried by a few units) or a distributed representation (spread across many units). Therefore, we quantified two complementary properties of the activations: the extent to which context sensitivity was concentrated in a small subset of units (akin to individual “context cell” neurons that code for which context is currently active), and the degree to which the currently active context was expressed as a pattern of activity across the hidden layer. These analyses were conceptually motivated by prior work distinguishing sparse and distributed coding strategies in hippocampal and connectionist models.^34^^,^^40^ The analysis was conducted using the hidden-layer activations during the final block of training.

**Figure 5:**
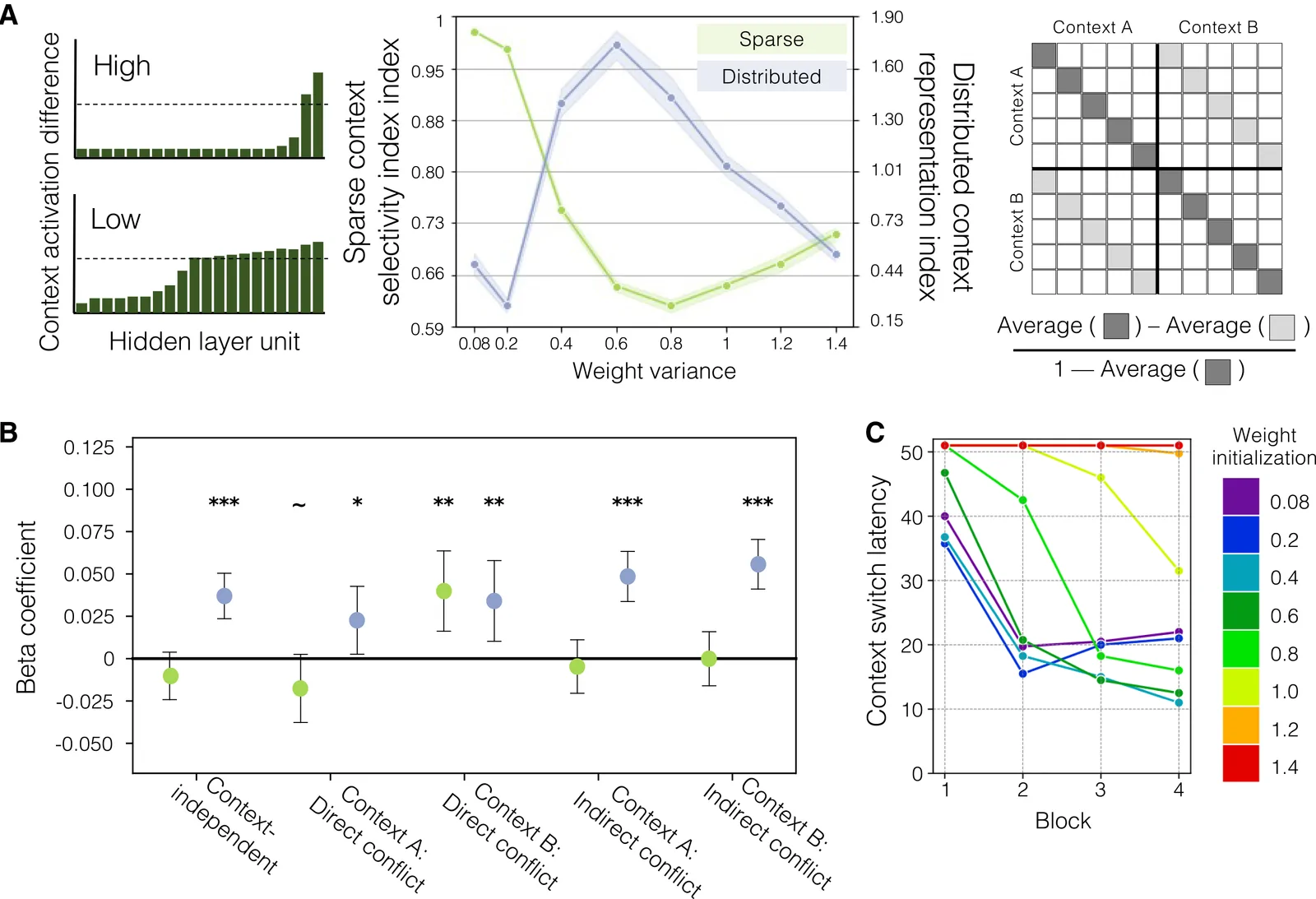
Neural network hidden layer task representation strategies (A) Schematic of computation of sparse context selectivity index (left) and distributed context representation index (right) plotted for each weight variance configuration (middle; *x* axis; mean ± SEM) in light green and blue, respectively. (B) Significance of beta coefficients (*y* axis) for linear regression analyses using sparse context selectivity (green) and distributed context representation (blue) indices to predict model performance for each 2AFC question category (*x* axis). ∗∗∗*p* < 0.001, ∗∗*p* < 0.01, ∗*p* < 0.05, ∼*p* < 0.1. (C) Context switch latency (*y* axis) visualized across learning phase blocks (*x* axis) for each weight initialization configuration (rainbow coloring). Failure to reflect a context switch is noted with a value of 51 (e.g., greater than duration of context exposure).

The *sparse context selectivity index* measures the proportion of hidden layer units that do not show significant activation differences between contexts. A higher sparse representation index therefore indicates that fewer units selectively encode a specific context, while a larger proportion of units are context-insensitive (Figure 5A). This measure of context sensitivity for each unit was calculated with a one-way ANOVA, comparing activations during exposure to Context A versus Context B in the final quarter of training (block 4). “Sparsity” is used here to refer to the distribution of context selectivity across units, not sparsity of overall activation. Fewer nodes with significant context sensitivity indicate that a limited number of hidden-layer units support the context-specific representations.

The *distributed context representation index* was derived from a representational similarity analysis of hidden layer activations for the first item of each pair, the item that carries the context-dependent association. The index compares the geometric distance (dissimilarity) of these representations within a context versus across contexts, with normalization based on within-context consistency so that more stable representations are given greater influence (Figure 5A, right plot). “Distributed” is used here to describe how context modulates population-level activation patterns across units, rather than the number of individually context-selective units. Higher values indicate that the same input is represented more distinctly across contexts, reflecting that context-dependent representations are distributed across the hidden layer.

We found that the low-variance models exhibited sparse context selectivity, indicating that context sensitivity was confined to a relatively small subset of units, whereas the moderate-variance models exhibited a more distributed representation of context, reflecting greater differentiation of representations across the hidden layer (Figure 5A, middle plot). High-variance models do not show strong evidence of either representation strategy. The 0.6 weight variance condition, which was the only weight condition to demonstrate significant direct-conflict 2AFC accuracy for both contexts (Figures 4C and 4D), exhibited the strongest evidence of the distributed context representation index of all models, with significantly higher values than all other weight initialization configurations (pairwise t-tests, all *p* < 0.006).

To understand how these representation strategies supported learning, we regressed 2AFC accuracy on the *z*-scored sparse context selectivity and distributed context representation indices in separate linear regression models. Each trained neural network model was treated as an individual sample. The low-variance models exhibited qualitatively asymmetric performance—near-ceiling accuracy for Context B but below-chance accuracy for Context A direct-conflict trials—reflecting a systematic bias toward the conflicting associate rather than a graded continuum of chance-level to high accuracy that the regression was intended to capture. Group-deletion analyses were conducted to measure the change in regression coefficient after excluding a given group rescaled by standard error of the coefficient in the full model. The analyses confirmed that the low-variance models (0.08 and 0.2) exerted substantially greater influence on regression coefficients (|Δβ| > 2.5; peaking at Δβ = −6.8) than all other regimes (|Δβ| < 1.7). Accordingly, the reported regression analyses (Figure 5B) excluded these two regimes and modeled the behavior of 300 neural networks (50 randomly initialized models for each of the six weight initialization conditions included in analysis).

The distributed context representation index was significantly associated with 2AFC performance on context-independent trials as well as direct- and indirect-conflict trials in both Context A and Context B (all *p* < 0.027). Critically, it was significantly associated with performance on direct-conflict trials in both contexts, the most stringent test of context-dependent learning (Context A, *β* = 0.023, *p* = 0.027; Context B, *β* = 0.034, *p* = 0.006). In contrast, the sparse context selectivity index was significantly associated only with Context B direct-conflict performance (β = 0.040, *p* = 0.001) and did not show a significant relationship with Context A direct-conflict performance (β = −0.020, *p* = 0.086).

### Context switch latency as a measure of online learning

To explain differences in context-dependent 2AFC performance, we examined how efficiently models adapted to context switches. We derived a context switch latency metric operationalized as the number of pairs the model processed after a context switch before perfectly predicting the paired associate of all remaining pairs in each 50-pair context exposure. This measure complements human RTs to perceptual judgments during learning while providing a more direct evaluation of prediction accuracy, as the model is trained to predict the next item in the sequence and RTs reflect facilitation from learned associations rather than directly indexing prediction accuracy. We found that, by the final block of training, the moderate-variance models exhibited faster switch latencies whereas the low-variance models adapted more slowly (Figure 5C). This inefficiency likely prevented the low-variance models from updating their internal state during the brief six-item exposure provided in each 2AFC trial, leaving them biased toward Context B despite evidence of retaining earlier Context A associations. Overall, these results indicate that the moderate-variance models achieve the best context-dependent learning because they retain associative knowledge across contexts and quickly adapt to context switches with support from a distributed representation of context across the hidden layer.

## Discussion

Given that statistical learning enables associative strengths to be incrementally updated over many exposures, it has been unclear whether it affords sufficient flexibility to adapt to changing associative contingencies in different contexts. The present results extend our understanding of when incidental learning of temporal regularities is possible via demonstration of context-dependent statistical learning under circumstances where contexts dynamically alternate, associations directly conflict, and no explicit instructions are provided. We found evidence for this in above-chance performance on the final 2AFC test as well as progressive RT speeding for predictable objects compared to unpredictable objects over the course of learning, which suggests that implicit learning mechanisms facilitated anticipatory behavior.^41^

Notably, explicitly signaling context with a colored border (Expt. 2) did not enhance context-dependent learning compared to when context was fully latent (Expt. 1). This may reflect greater influence of local temporal context (e.g., recent sequence history, which was available during both learning and retrieval) over environmental cues or disruption of implicit learning mechanisms by promoting a more deliberate strategy. Indeed, recent work suggests that states of reduced executive control, such as mind wandering, can enhance statistical learning relative to focused on-task states,^42^ implying that exogenously focusing attention via explicit cues may be counterproductive for this type of incidental learning. However, given that the border cue changed color only every few minutes and its relevance was not explicitly conveyed, it is also possible that this visual cueing of context may have been too subtle to provide a performance advantage; a more salient context signal might have produced different effects.

To the best of our knowledge, this study provides the first demonstration of context-dependent statistical learning in the visual domain under circumstances where contexts dynamically alternate, associations directly conflict, and no explicit learning instructions are provided. However, prior research is limited and the findings equivocal. Empirical studies with auditory stimuli have been unable to find learning of both contexts unless participants were provided with explicit instructions or salient context cues.^17^^,^^18^ Our success in the visual domain supports accounts suggesting that statistical learning mechanisms may be modality-specific rather than fully domain-general,^43^ though modality alone is unlikely to fully explain these discrepancies. For example, blocked exposure to overlapping structures in auditory paradigms without explicit context cues yielded significant learning of only the first structure^18^ possibly due to reduced sampling of the environment once regularities were established, as indexed by a reduction in neural activity.^44^ In contrast, the interleaved exposure of the present study (i.e., alternating back-and-forth between the two contexts many times) may have promoted continuous updating of context representations, enabling learning of both structures even without explicit cues. With that said, evidence of context-dependent learning with blocked exposure has been reported in the visual domain,^19^ but task design choices of self-paced exposure and explicit instructions to look for patterns in which shapes tended to follow each other may have encouraged strategic encoding and obscured whether the learning can be attributed to the exposure curriculum. Such design choices contrast with the present study, where shorter, fixed-duration stimulus presentations served to minimize possibilities for strategic encoding and support the passive, implicit learning that is thought to characterize statistical learning.^45^ Future work should systematically manipulate task design factors to determine how structure, exposure dynamics, and contextual cues shape context-dependent statistical learning.

The present findings build on prior work on second-order conditional (SOC) sequence learning, which has demonstrated that learners can extract higher-order temporal dependencies in which predictability depends on combinations of preceding elements rather than simple pairwise transitions.^41^^,^^46^ Recent work further suggests that exposure to SOC structure can shape subsequent performance and subjective sensitivity to sequence regularities even when explicit knowledge is limited.^47^ Although the surface structure of these tasks differs from the present paradigm, both lines of work underscore how context-sensitive behavior can emerge from the integration of temporal regularities over experience, without requiring explicit contextual signals.

We used a neural network modeling approach to inform hypotheses about how the human brain might support such learning. These models were optimized to predict the next object in the sequence. While our human participants engaged in a cover task requiring simple ×/+ perceptual judgments, we assume that they were implicitly forming predictions about upcoming stimuli. Therefore, the models’ predictive framework captures a core computational goal that the human learners pursue implicitly: anticipating future input based on recent experiences.^1^

The GRU’s gating architecture may support its successful context-dependent learning by enabling the model to manage conflicting associations based on retaining relevant information while filtering out noise. This computational function parallels how learning systems manage interference between new and old memories.^48^ Although GRU models are not intended as models of biological mechanisms, the update and reset gates can be viewed as implementing this balance by regulating the extent to which incoming input modifies the current state versus preserving learned representations from past experience. At a functional level, this is analogous to proposed mechanisms in the brain that mediate the trade-off between updating and stability, including interactions between the hippocampus and neocortex.^33^ It also parallels neuromodulatory systems where prediction error signals (e.g., dopamine release) convey the need to update internal representations, prompting a reassessment of context and a switch in behavioral strategy.^49^ Such mechanisms have been hypothesized to facilitate segmentation of continuous experiences and recalibration of predictions,^50^ which may be relevant for context-dependent temporal associative learning and motivate hypotheses for future work examining parallels between biological systems and computational models.

Prior neural network modeling of context-dependent learning has imbued neural networks with specialized architecture to facilitate latent cause inference^27^^,^^28^^,^^51^ or has explicitly provided unambiguous context information in model input.^21^^,^^26^ For example, Smith and colleagues^21^ effectively demonstrated that recurrent networks can track temporal structure across multiple timescales within explicitly signaled contexts in a statistical learning paradigm instantiated as games that share response choices. However, these studies bypass the question of how a sense of context might emerge organically from exposure alone to disambiguate overlapping task structure. Additionally, they introduce assumptions that are arguably biologically implausible, such as constant context monitoring and perfectly reliable context cues.^5^ Here, we more directly focus on latent context discovery by exploring how weight initialization affects learning dynamics. Since network weights are adjusted throughout training to minimize loss, their initial configuration acts as a key driver of convergence.^52^ Prior work suggests that higher initial weight magnitudes bias models toward “lazy” solutions, involving rapid solution convergence with unstructured representations, while smaller magnitudes support “rich” solutions that exhibit more structured learning, albeit at a slower pace.^26^^,^^32^^,^^39^

Indeed, increasing the variance of the uniform distribution used to initialize model weights to a moderate range facilitated successful context-dependent 2AFC performance. These models showed the strongest distributed context representation index, indicating that identical cue objects resulted in distinct hidden layer states depending on context. This is consistent with studies suggesting that high dimensional codes afforded by mixed selectivity in prefrontal cortex neurons allow for more flexibility and rapid adaptation to new tasks.^40^^,^^53^ The successful distributed context coding strategy where identical model input is represented differently when processed in different contexts is consistent with reports of the hippocampus integrating contextual information into stimulus representations.^54^^,^^55^ Furthermore, the hippocampus supports the rapid learning of temporal associations.^36^^,^^56^ In our model, however, item representations are already non-overlapping (i.e., coded as one-hot vectors at the input stage) and the training objective directly supports learning of temporal associations, such that successful next-item prediction requires representing latent context to disambiguate conflicting associations. Taken together, these parallels suggest that the moderate-variance GRU models are capturing representational properties of both higher-level contextual encoding and lower-level temporal associations, though we do not intend this as a direct mapping onto specific hippocampal or neocortical pathways or mechanisms.

The variance of weight initialization may be interpreted as shaping the GRU’s inductive bias: the assumptions the model makes about the structure of the environment, particularly regarding the presence and separability of underlying contexts. Low initial weight variance appeared to bias the model toward rigid representations that emphasize recently experienced associations and failed to recover earlier learned patterns following context shifts. On the other end, high initial weight variance produced overly flexible representations that failed to consolidate stable structure. Our analyses suggest an optimal intermediate range of initial weight magnitudes, where models were sufficiently flexible to distinguish between contexts yet structured enough to preserve associations within each context and avoid catastrophic interference. Accordingly, these effects are best understood as emergent inductive biases shaped by properties of training dynamics and initialization, which may provide insight into how learning systems come to represent and segregate latent contexts, whether biological or artificial. Future work could assess the extent to which similar learning dynamics arise across architectures and task demands and whether manipulating network hyperparameters, such as learning rate and number of hidden layers, consistently shape the balance between plasticity and stability in context-dependent learning.

To better understand why the moderate-variance models succeeded, it is informative to examine the limitations of the low-variance models since low-variance initialization is commonly used as the default setting for neural networks. Performance on indirect-conflict trials indicates that the low-variance models acquire associative knowledge of context-dependent pairs. However, failure on the Context A direct-conflict trials demonstrates that these models do not successfully use context information to disambiguate competing associations. That is, these models demonstrate learning of which associations are plausible but not which association is correct given the current context. Relatedly, these low-variance models exhibited slower context switching than moderate-variance models. As a result, the brief context exposure sequences preceding each 2AFC decision may have provided insufficient evidence to trigger a shift in their internal state, leaving the models biased toward the most recently learned context.

Mirroring the diversity of these computational profiles, humans also exhibited considerable variability. Although performance on context-dependent trials was significantly above-chance at the group level, some participants exhibited little or no learning (akin to the high-weight models) while others showed stronger learning of one of the contexts (similar to the low-weight models). Just as some GRU models required more exposure to learn both sets of associations, certain individuals may also need more input to reach stable learning. This pattern is consistent with recent work demonstrating that humans and neural networks share parallel trade-offs between knowledge transfer and interference, with individual differences mapping onto “lazy” and “rich” representational regimes in neural networks.^57^ In our model, weight initialization may instantiate a similar axis of inductive bias, shaping representational strategies and learning outcomes. A promising future direction is to identify model parameters that predict how quickly a learner converges on context-dependent associations, potentially linking such parameters to developmental changes in learning efficiency.^58^

Variability may also arise from the structure of the learning environment itself. Among the task design factors that may shape both human and model performance, context exposure length and the frequency of context switching represent a particularly informative dimension. Prior work has shown that recurrent neural networks often struggle to capture curriculum effects observed in humans, particularly cases in which blocked exposure facilitates context-dependent learning relative to interleaved exposure.^59^ The ability of the moderate-variance GRU model to achieve comparable performance to humans in the present task is therefore encouraging, and future work should test how longer context exposures influence learning of both humans and models.

Overall, our findings demonstrate that humans can spontaneously resolve conflicting, context-dependent associations from passive exposure alone—even in the absence of explicit instructions, self-pacing, feedback, or contextual cues. The finding that explicit signaling offered no advantage over entirely latent context exposure further highlights the robustness of this incidental learning mechanism, suggesting that temporal statistics alone are sufficient to drive contextual inference. Our neural network modeling provides a mechanistic account for this capacity, showing that successful adaptation relies on the emergence of distributed representations that are influenced by weight initialization parameters, which we believe are a reasonable proxy for humans’ inductive biases. This representational strategy not only maintains information from multiple contexts, even when the associative structure directly conflicts across contexts, but also quickly accommodates context changes. These findings suggest that the human brain may rely on similar mechanisms to flexibly manage latent contextual shifts and support adaptive prediction in dynamic environments.

### Limitations of the study

The generalizability of our findings is limited by several factors that will require additional work to resolve. As mentioned above, the black/white border used to signal context in experiment 2 may have been too perceptually subtle to serve as a meaningful contextual signal, leaving open the question of whether a more salient context cue would enhance context-dependent learning beyond what is achieved through sequence statistics alone. For instance, if the objects were presented against a backdrop of complex scenes that periodically alternated, perhaps this would have been more heavily attended to throughout the exposure phase and integrated with the object identity information to foster contextually gated associative predictions. Alternatively, it is possible that accentuating the context salience might encourage participants to use a more explicit hypothesis-testing strategy to discover the relevance of the changing contexts, and this could potentially push participants toward episodic learning rather than more incrementally acquired statistical learning. It would also be worthwhile to explore whether participants could achieve even stronger context-dependent learning if we increased the amount of exposure beyond the four blocks (of 200 pairs each) used in our study. Our reaction time data indicated online learning that progressively increased across the four blocks, and it seems likely that with additional exposure training, behavioral speeding to predictable objects would be accentuated. That said, RTs serve as an indirect proxy for associative knowledge, reflecting facilitation from learned predictions rather than directly indexing prediction accuracy. Future work employing more direct measures of associative strength during learning (e.g., inserting intermittent “what comes next” 2AFC probe trials) would strengthen conclusions about the time course of context-dependent statistical learning in humans. Furthermore, because our 2AFC test always occurred immediately after conclusion of the exposure phase, we do not yet know how long the context-dependent associative learning achieved by our participants would last. It would be interesting to test whether this knowledge can be retained for hours, days, or weeks.

Regarding the neural network models, although GRU networks are not intended as direct models of neural mechanisms and their gating operations do not map cleanly onto known biophysical processes, they have successfully captured key features of human learning behavior and generated testable hypotheses about the representational strategies that may support context-dependent statistical learning (e.g., emergence of highly distributed representations of context). The biological parallels drawn here are therefore best understood as functional analogies that motivate future empirical investigation rather than claims about specific neural implementations. Finally, the modeling framework embeds the assumption that statistical learning is achieved through sequential, next-item prediction, consistent with transition probability tracking accounts.^11^ Alternative accounts such as chunking have also been proposed,^60^^,^^61^ and the present approach is not designed to adjudicate between them.

## Resource availability

### Lead contact

Requests for further information should be directed to and will be fulfilled by the lead contact, Jesse Rissman (rissman@psych.ucla.edu).

### Materials availability

Task scripts are available on GitHub upon publication. The stimuli used in this study were drawn from previously published resources.^62^^,^^63^

### Data and code availability

• Behavioral data are available on Open Science Framework: https://osf.io/7v38d/files/osfstorage.
• Analysis scripts are provided on GitHub: https://github.com/fcpeck/statistical_learning.
• Any additional information needed to reanalyze the data is available from the lead contact upon request.

## Acknowledgments

F.C.P. was supported by the 10.13039/100023581National Science Foundation Graduate Research Fellowship Program under grant nos. DGE-2034835 and DGE-2444110 and the UCLA Dissertation Year Award.

## Author contributions

Conceptualization, F.C.P., J.R., and H.L.; methodology, F.C.P., J.R., and H.L.; software, F.C.P.; formal analysis, F.C.P.; data curation, F.C.P.; visualization, F.C.P.; supervision, J.R. and H.L.; writing – original draft, F.C.P.; writing – review and editing, F.C.P., H.L., and J.R.

## Declaration of interests

The authors declare no competing interests.

## STAR★Methods

### Key resources table

**Table:** 

REAGENT or RESOURCE	SOURCE	IDENTIFIER
**Deposited data**		

Data	This paper	https://osf.io/7v38d/files/osfstorage

**Software and algorithms**		

Python	Python Software Foundation	Version 3.8.8; www.python.org
PsychoPy	Open Science Tools Ltd.	Version 2024.2.4; www.psychopy.org
PyTorch	PyTorch Team	Version 2.0.1; www.pytorch.org
All code	This paper	https://github.com/fcpeck/statistical_learning

### Experimental model and study participant details

Participants were recruited via the UCLA Psychology Department subject pool and completed the experiment in-person for course credit. All participants provided informed consent in accordance with protocols approved by the UCLA Institutional Review Board (IRB#22-001719). Inclusion criteria were: age between 18 and 40 years, native English speaker, and normal or corrected-to-normal vision with contacts (no glasses). Our goal was to obtain usable data from 50 participants for each of the two experiments (Expt. 1: Unsignaled context; Expt. 2: Signaled context), so data collection continued until our data quality thresholds were met: 90% of trials responded to and 85% accuracy on trials during the learning phase. These inclusion criteria were enforced to ensure that data analyses focused on participants who were engaged during the learning phase of the experiment. Our final sample included 50 participants for Expt. 1 (33 F/17 M; mean age = 20.5 years) and 50 different participants for Expt. 2 (40 F/8 M/2 Non-Binary; mean age = 20.0 years).

### Method details

#### Apparatus and stimuli

The experiment was coded and run with PsychoPy version 2024.2.4^64^ on a Mac Mini. Stimuli were displayed on a DELL P2422HE monitor with 1920 by 1080 pixel resolution and screen size of 23.8 inches, which participants viewed from a fixed distance with their head stabilized with a forehead and chin rest. An EyeLink 1000 eye tracker (SR Research) captured gaze location while participants completed the experiment, but eye tracking data are not reported here. Experiment stimuli were drawn from a set of objects created using Blender 2.48.^62^^,^^63^ The stimuli were visually distinct in terms of shape and color and were novel to participants. Images were resized to be 350 pixels wide. A small “×” or “+” symbol was subtly embedded onto each object using slight color contrast such that the mark was visible but did not obstruct recognition of object shape.

#### Learning phase

In the first phase of the experiment, participants were exposed to a sequence of objects presented individually. The objects were presented in four different locations on the screen with a width of 350 pixels and centered 300 pixels above, below, right, and left of the center of a gray screen. At each object presentation, the three positions not occupied by the current object were filled with phase-scrambled versions of other objects cropped into circles with diameter of 300 pixels (visualized in *SI Appendix,* Figure S1). The experimental manipulation of object location was included to enable potential analyses of spatial location-based learning as indexed by anticipatory eye movements. However, because the eye tracking data did not yield clear or interpretable effects, analyses focus on object identity and omit spatial position from further consideration, as well as from the task depiction in Figure 1. Importantly, each object was always presented in the same spatial location across both Context A and Context B, such that spatial position was not diagnostic of context or paired associations and could not serve as a confounding context cue. Before beginning the learning phase, participants were instructed that parts of the sequence might become familiar over time and that they would later be asked questions about the objects they had seen.

Unbeknownst to the participants, the objects were organized into two sets (or contexts) of five pairs of objects. The same object set was used for all participants but objects were randomly assigned to each pair position, and each object maintained either first-of-pair (item 1) or second-of-pair (item 2) position in the pair across contexts. One pair was context-independent, meaning the same two objects were paired in both contexts. The other four pairs were context-dependent. Three of these pairs consisted of the same set of six objects across both contexts, but the second item associated with each first item was dependent on context. For example, Object X is paired with Object Y in Context A but with Object Z in Context B. The last context-dependent pair shared the same first object across both contexts but the paired second object was specific to each context (e.g., only appeared in that context and not the other). In this way, these four context-dependent pairs shared a first item across contexts but the second item of each pair was dependent on context. In total, 11 unique items were used to instantiate the five pairs in each context.

The learning phase was organized into four blocks, with short breaks between blocks. Within each block, there were two exposures to Context A and two to Context B. Each exposure consisted of 50 pairs (100 objects), with each of the five pairs presented 10 times. Contexts alternated throughout the block, resulting in three context switches per block. During this phase, participants were tasked with responding to whether the object onscreen was marked with an “×” or “+”. Therefore, reaction time to the perceptual question could be evaluated as an online measure of pair structure learning. Objects were presented for 1200 ms with a 450 ms interstimulus interval.

The two experiments differed with respect to whether context was Unsignaled (Expt. 1) or Signaled (Expt. 2) with a border around the objects that was white or black depending on the context.

#### Two-alternative forced choice (2AFC) task

In the first of three test tasks immediately following this learning phase, participants completed a two-alternative forced choice (2AFC) task. Because the object associations were dependent on active context for all but the one context-independent pair, on each 2AFC trial participants were presented with a sequence of seven objects (consisting of three pairs from one of the contexts and the first item of the test pair) before being presented with two side-by-side alternatives as to which object they think should come next (one was the correct paired associate of the test pair and the other was a lure). Objects were presented with the same timing as used during the learning phase in the sequence, and participants were given unlimited time to make a choice between target and lure. Participants completed a total of 54 questions: 6 of these questions evaluated the context-independent pair, while 48 questions evaluated context-dependent associations. The 48 questions probing context-dependent associations could either feature a lure object that was the correct paired associate in the other context (direct-conflict; 16 questions), or a lure that was any other item (indirect-conflict; 32 questions). The “×” and “+” markings were removed from the objects to make clear that participants no longer were required to respond to the perceptual question. For the Unsignaled experiment, no explicit context cues were provided; for the Signaled experiment, the border around the objects was colored white or black on each trial to cue contexts. After making each 2AFC judgment, participants were prompted to rate their confidence in their decision from 1 to 4.

#### Structure knowledge probe

After completing all 2AFC trials, participants were prompted to answer some questions about what they learned during the experiment. First, they were asked to respond yes or no to whether they observed any predictable patterns in the experiment. Second, they were asked to describe any patterns they observed in the sequence. Third, they were asked to describe any rules that governed which object would come next in the sequence. The idea was to use progressively more straightforward prompts to determine how much of the knowledge was explicit.

#### Pair reconstruction task

The last task allowed participants to demonstrate explicit knowledge of pairs. Participants were presented with a bank of all 11 objects at the top of the screen and provided with 20 sets of two empty squares side-by-side presented in 4 rows of 5 columns. Participants were instructed to organize the objects into related pairs by placing one object in each square of a pair, with the left and right positions corresponding to the first and second items in the pair. Participants were told that each item could be used more than once and that they did not have to fill out all of the pairs.

#### Neural network architecture

Recurrent neural network models with gated recurrent units were implemented with PyTorch v2.0.1.^65^ Such models have previously been used to explore context-dependent associative learning from sequences.^21^^,^^26^ Each model had the same architecture: an 11-node input layer with dimensionality of 11 (equal to the number of objects included in the study), a hidden layer of 150 nodes (GRU performance with different hidden layer sizes presented in Methods S4 and Figure S6), and an 11-D output layer again to match dimensionality of one-hot object vectors. The learning rate was held constant at 0.001, and model weights were updated after each training sample using the Adam algorithm of gradient descent and cross entropy loss. Default parameters were used unless otherwise noted.

#### Training

A unique 1600-object sequence was generated for each model in the same way as for human participants. Each neural network received one object at a time and was trained to predict the identity of the next object in the sequence. Although the sequence was constructed using embedded object pairs, models received no information about this underlying structure. That is, the model made predictions at every time step (1599 samples for the 1600-object sequence) and had no awareness of pair boundaries. The hidden state reset between blocks (every 400 samples) to emulate the breaks taken by human participants.

#### 2AFC task

After learning, model weights were frozen, and the models were evaluated using a 2AFC test designed to mirror the testing procedure of the human participants. A unique set of 2AFC test questions was generated for each model in the same way as for human participants. Before each trial, hidden layer activity was reset to zero. Then, a sequence of three pairs from one of the contexts was presented as the hidden state evolved, allowing the model to infer the active context based on the sequence. Finally, the first object of the test pair was inputted, and the model’s prediction of the ensuing item was evaluated.

#### Weight initialization conditions

We tested the GRU’s ability to learn the task as the variance of the uniform distribution used to initialize the hidden layer’s weights was increased. The uniform distribution was centered at zero with positive and negative bounds of 0.08 (default for PyTorch with 150 nodes), 0.2, 0.4, 0.6, 0.8, 1.0, 1.2, and 1.4. Fifty independent GRU models with different weight initialization randomizations were trained and tested, and learning measures across these models were averaged to ensure robust performance estimates of each weight initialization category.

### Quantification and statistical analysis

Significance levels are represented as “∼” for *p* < 0.1, “∗” for *p* < 0.05, “∗∗” for *p* < 0.01, and “∗∗∗” for *p* < 0.001. Bar height reflects group mean. Error is SEM across figures.

#### Behavioral performance analysis: Human participants

2AFC test performance was evaluated using accuracy. Accuracy was computed separately for Context A and Context B (24 questions each) and for context-independent trials (6 questions). Context-dependent trials were further subdivided into direct-conflict trials (8 questions for each Context) and indirect-conflict trials (16 questions). To assess experimental effects, 2AFC accuracy was analyzed using mixed-design analyses of variance (ANOVAs) with Experiment (Signaled vs. Unsignaled) as a between-subjects factor and context-dependence (context-dependent vs. context-independent trials) as a within-subject factor. Separate ANOVAs were also conducted for direct- and indirect-conflict trials with Context as a within-subject factor and Experiment as a between-subjects factor. A Bayesian equivalence test was conducted to evaluate whether overall context-dependent performance differed between experiments. Posterior distributions of the mean difference in 2AFC accuracy between experiments were estimated using the PyMC package. Equivalence was assessed using a predefined region of practical equivalence (ROPE) of [–5%, 5%], and the proportion of posterior mass falling within this interval was used to quantify evidence for equivalence.

Online learning during the learning phase was assessed using RTs on the perceptual judgment task. An anticipation score was computed for each block as the difference between mean RT to the first item of each pair and mean RT to the second item (RT(item1) – RT(item2)), such that positive values indicate faster responses to predictable second items. To assess changes in learning over time, a linear trend across blocks was tested using contrast weights [-3, -1, 1, 3], with the resulting contrast values evaluated against zero using two-tailed one-sample *t*-tests. These analyses were conducted separately for each experiment and for context-independent and context-dependent pairs. To compare learning trajectories across experiments, mixed-design ANOVAs were conducted separately for context-independent and context-dependent anticipation scores with Experiment as a between-subjects factor and Block as a within-subject factor. This analysis tested for differences in the rate and magnitude of online learning across conditions.

To evaluate the Structure Knowledge Probe, participants’ written responses were manually scored as a binary outcome based on whether they articulated explicit awareness of the temporal pair structure. This measure did not account for knowledge of the dual-context structure.

For the Pair Reconstruction Task, performance was evaluated against an analytical baseline of chance because participants could report a variable number of pairs (k = 1–20). With 11 unique objects, choosing 2 objects without replacement yields 110 possible unique permutations. Across both contexts, there were 9 valid target pairs (including one context-independent pair). Thus, the baseline probability of randomly guessing a correct pair on any given trial was fixed at 9/110, or 8.2%

#### Behavioral performance analysis: Neural network models

Model performance was evaluated using the same 2AFC procedure administered to human participants. Accuracy for each trial was computed as correct when a higher probability was assigned to the correct object than to the lure. Accuracy was computed separately for Context A and Context B (24 trials each), with context-dependent trials further subdivided into direct-conflict (8 trials per context) and indirect-conflict (16 trials per context) conditions, as well as for context-independent trials (6 trials). Performance was evaluated across weight initialization conditions and averaged across 50 independent trained models per condition. Above-chance performance was assessed by comparing accuracy to 50% chance using two-tailed one-sample *t*-tests conducted separately for each condition.

Correspondence between model and human performance was computed as the absolute difference between model and human 2AFC accuracy on direct-conflict trials for Context A and Context B for each weight initialization condition. Model configurations were evaluated based on two criteria: (i) significant above-chance performance on direct-conflict trials in both contexts, and (ii) minimal deviation from human performance on these trials.

To quantify adaptation to context changes, we defined a switch latency measure as the number of first-of-pair (item 1) inputs processed following a context switch before the model achieved perfect prediction accuracy on all remaining item 1 samples within that context exposure. Item 1 inputs were used because they index the within-pair transition prediction. Switch latency was computed for each of the 16 context exposures (8 per context) and averaged within blocks (4 exposures per block), yielding a single value per block. For each weight initialization condition, switch latency was averaged across 50 independently trained models.

#### Neural network hidden layer representational analyses

To quantify how context information was represented in the hidden layer, we computed two complementary indices: a sparse context selectivity index and a distributed context representation index. These measures capture whether representation of context is localized to a subset of units or expressed as distinct patterns of activity across the hidden layer. All analyses were conducted on hidden layer activations from the final quarter of training (block 4).

The sparse context selectivity index quantified the extent to which context sensitivity was confined to a subset of hidden layer units. For each of the 150 hidden units, a one-way analysis of variance (ANOVA) was performed to compare activation values during Context A and Context B. A Bonferroni correction was applied within each model to account for the 150 comparisons performed (α = 0.05/150 = 0.00033, corresponding to F_crit_(1,398) = 12.75). Units not exceeding this threshold were classified as non-selective. The sparse representation index was defined as the proportion of units that did not show a significant difference in activation between contexts, such that higher values indicate greater sparsity of context representation.

The distributed context representation index was computed using a representational similarity analysis (RSA;^66^) on the hidden layer activations following input of the first item of each pair (capturing the context-dependent prediction) during the final quarter of training (block 4). This comprised a total of 200 hidden state activity patterns (100 per context). These samples were divided into two split-halves, each containing 10 samples for each of the five pairs per context evenly drawn from the first and second halves of each exposure. Activation was averaged across instances of each object within each context for each hidden layer node. Pearson correlation coefficients were computed between all pairs of object representations across split halves, yielding an RSA matrix. Within-context similarity was defined as correlations between representations of objects within the same context, and between-context similarity as correlations between representations of the same objects across contexts. The distributed context representation index was defined as the difference between mean within-context and between-context similarity, normalized by (1 − mean within-context similarity) to penalize models with lower within-context similarity that would reflect noisy or unstable object representations. Higher values indicate greater differentiation of object representations across contexts.

Linear regression models were used to evaluate how the sparse context selectivity and distributed context representation indices related to 2AFC accuracy. Separate regressions were fit for each representation index. Outcome variables were context-independent accuracy and direct-conflict and indirect-conflict accuracy for both Context A and Context B. Predictors were z-scored prior to analyses to enable comparison of regression coefficients. Each trained neural network was treated as an individual observation (eight weight variance conditions x 50 randomly initialized models per condition = 400 models total). To assess influence of weight variance initialization conditions on regression estimates, a group-deletion analysis was performed in which models from each weight variance initialization condition were iteratively excluded and the model refit. Influence was quantified using a DFBETA-like metric (Δβ), defined as the change in the regression coefficient after exclusion of a group, scaled by the standard error of the coefficient in the full model. Weight initialization conditions exhibiting outsize influence on regression coefficients (|Δβ| > 2.5) were excluded from subsequent analyses. Regression analyses were conducted on a final sample of six weight variance conditions (*n* = 300 models).
