# Supplementary material for: Spontaneous emergence of context-dependent statistical learning in humans and neural networks
Source: iScience. 2026 Aug 12;29(8):116640. doi: 10.1016/j.isci.2026.116640 (PMC13523841; doi:10.1016/j.isci.2026.116640)
Supplement: Supplementary file 1 [file mmc1.pdf]

iScience, Volume *29*

Supplemental information

Spontaneous emergence of context-dependent statistical learning in humans and neural networks

Fleming C. Peck, Hongjing Lu, and Jesse Rissman

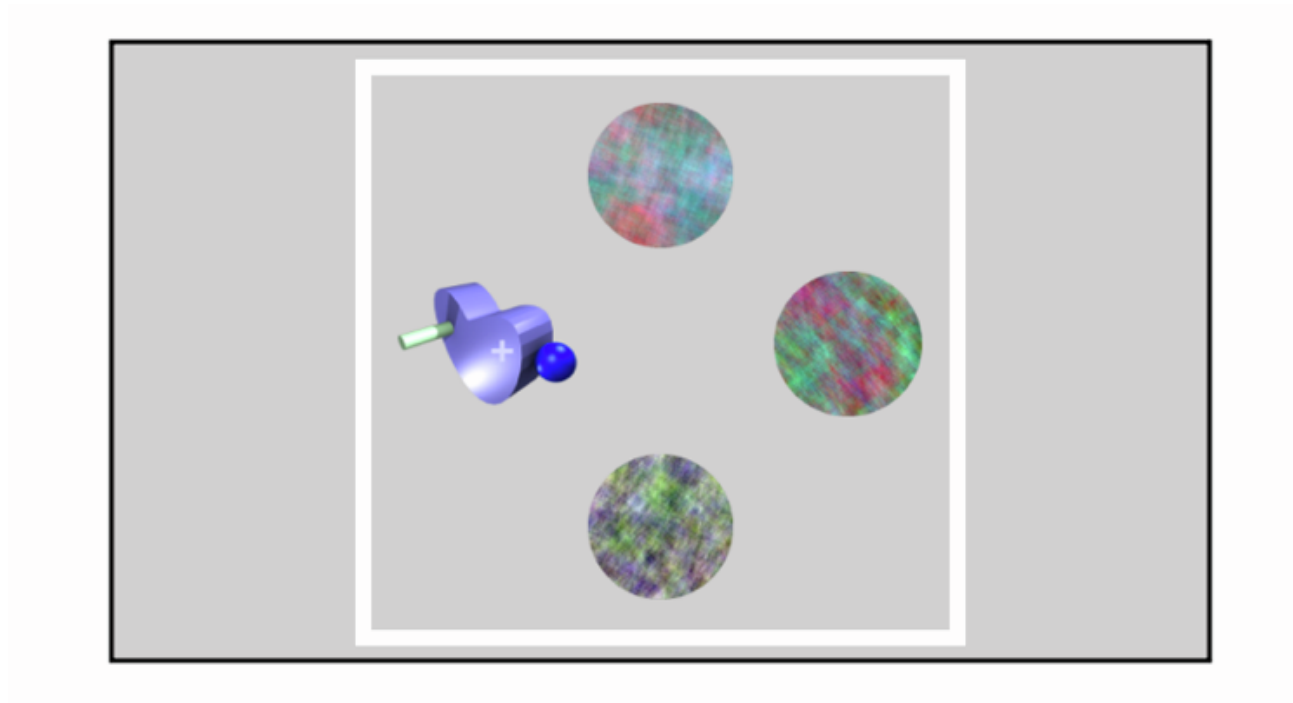

Figure S1. Visualization of stimulus presentation during learning phase.
Each trial of the learning phase featured four stimuli arranged as depicted, with one object of interest (on which participants needed to make an × /+ judgment) and three circular phase-scrambled objects presented in the remaining positions. A black or white border was present during Expt. 2. Visualization of objects and border is to scale.

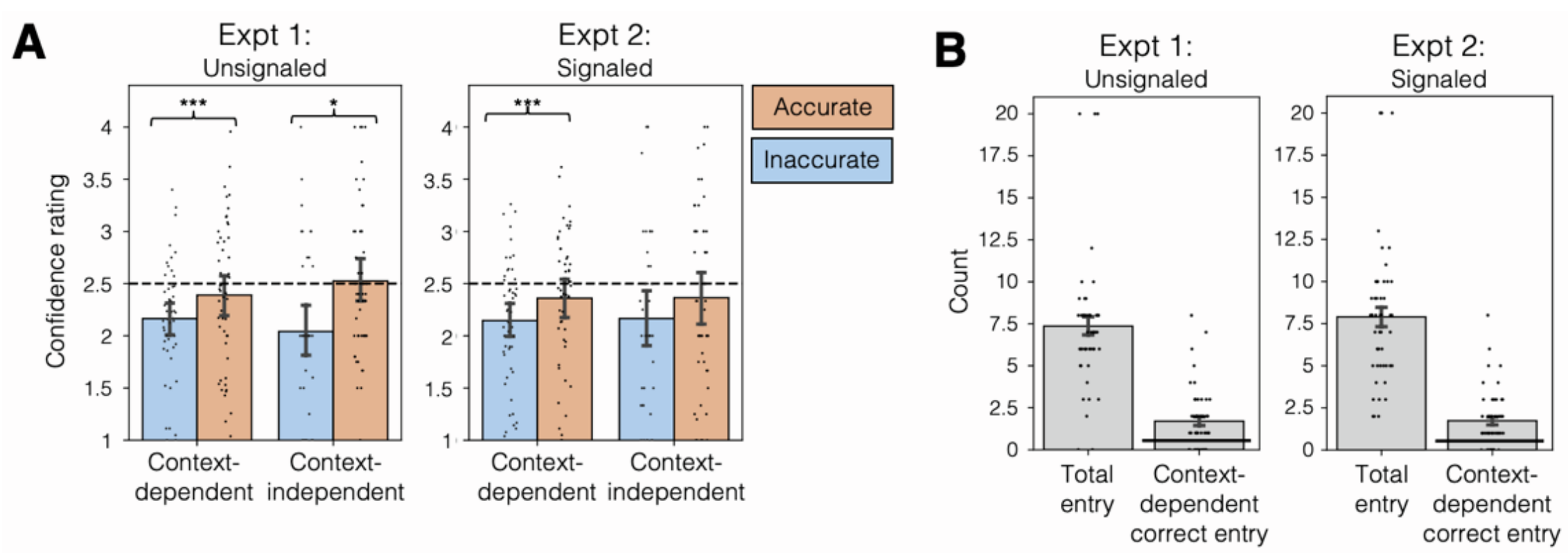

Figure S2. Metacognitive awareness assessment results.
(A) Average 2AFC confidence rating for context-dependent (left) and context-dependent (right) questions for accurate (pink) and inaccurate (blue) question responses. Horizontal dashed line indicates midpoint of the confidence scale, and error bars reflect SEM. *(B)* Pair Reconstruction Task performance: bar height reflects group average number of total pairs reported (left) and correct context-independent pairs reported (right). Horizontal line reflects chance performance based on Monte Carlo simulations; error bars reflect SEM.

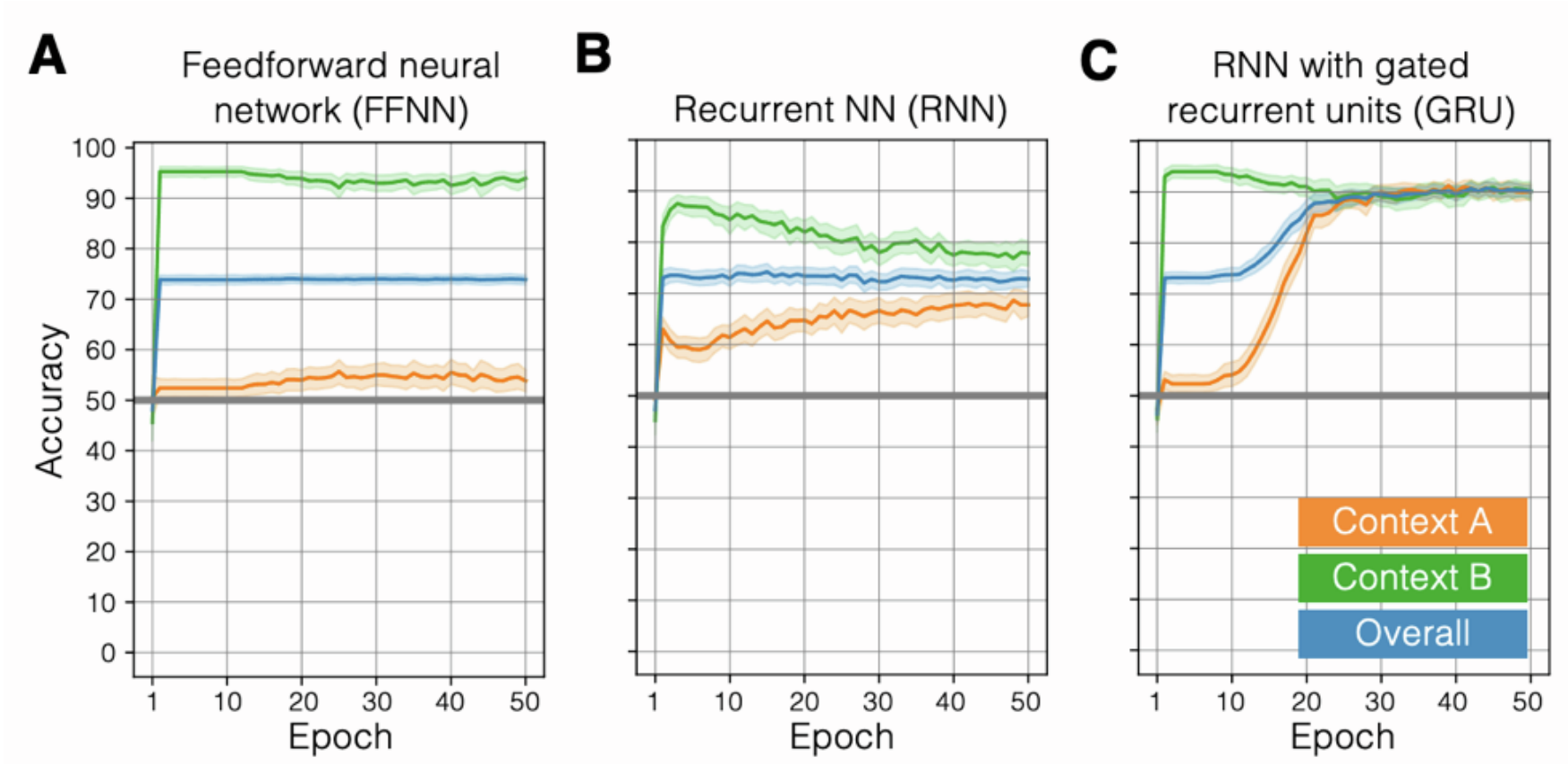

Figure S3. 2AFC task performance for three neural network model classes.
(A-C) 2AFC performance accuracy on context-dependent questions averaged for 50 individual models for each architecture after each of 50 epochs of training; accuracy is plotted separately for Context A (orange), Context B (green), and both contexts combined (blue) for each model class: *(A)* simple feedforward neural network (FFNN), *(B)* vanilla recurrent neural network (RNN), and *(C)* recurrent neural network with gated recurrent units (GRU). All models achieved perfect accuracy on context-independent questions after the first epoch (not pictured).

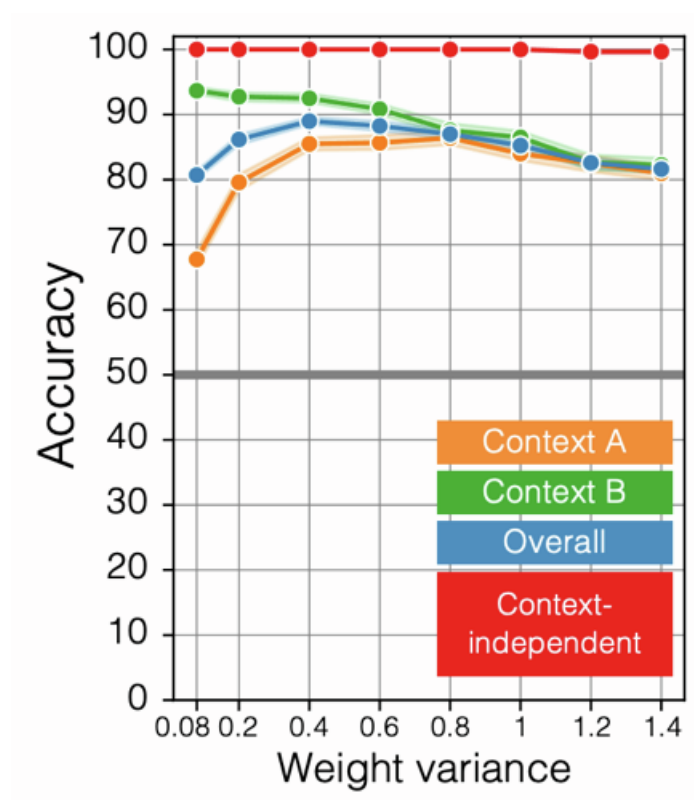

Figure S4. 2AFC performance with perceptual object embeddings.
2AFC accuracy (y-axis) on context-dependent test trials for GRU models with weights initialized with increasing variance along the x-axis color-coded by question category.

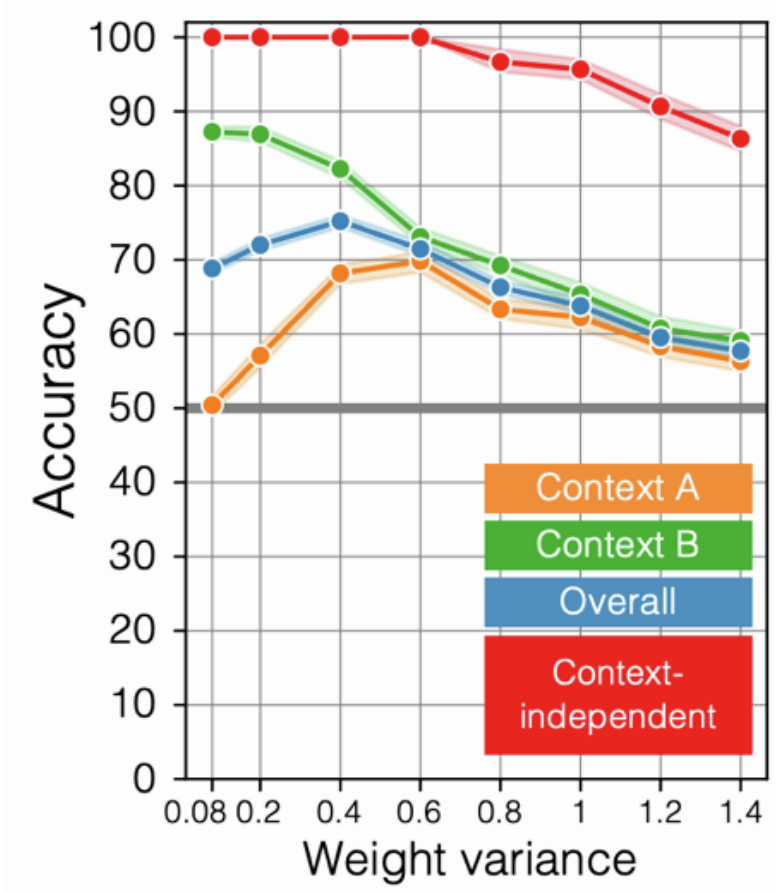

Figure S5. 2AFC performance with no context-specific objects measured by weight variance. 2AFC accuracy (y-axis) on context-dependent test trials for GRU models with weights initialized with increasing variance along the x-axis color-coded by question category.

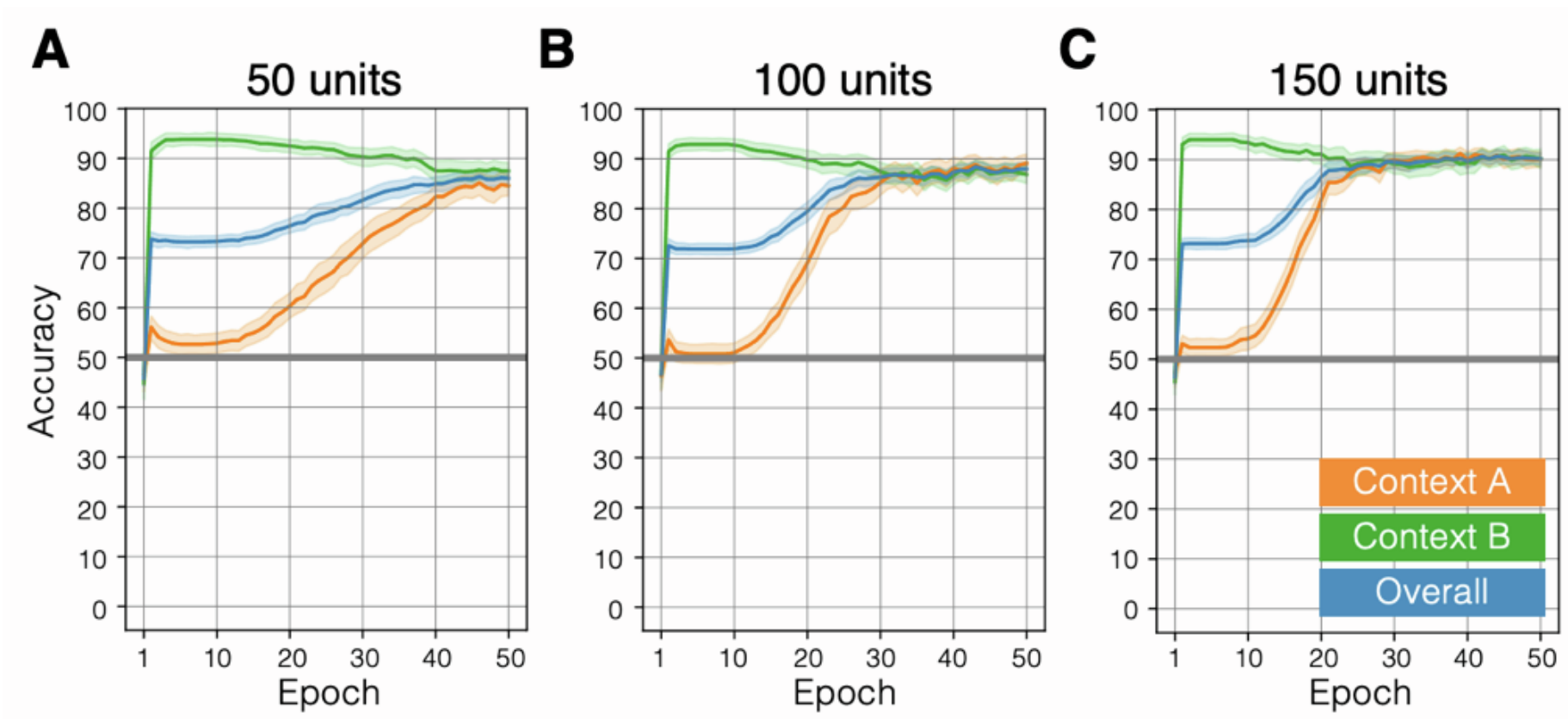

Figure S6. 2AFC task performance for GRU models with varying hidden layer sizes. *(A-C)* 2AFC performance accuracy on context-dependent questions averaged for 50 individual models for each architecture after each epoch of training for Context A (orange), Context B (green), and both contexts combined (blue) for GRU models with *(A)* 50 hidden layer units, *(B)* 100 hidden layer units, and *(C)* 150 hidden layer units.

Table S1. Reaction times (mean ± standard deviation) in milliseconds by block for context-dependent pair objects. Item 1 is the first, unpredictable element of each pair; Item 2 is the second, predictable element informed by the associative expectation.

	Expt 1: Unsignaled		Expt 2: Signaled	
	Item 1	Item 2	Item 1	Item 2
Block 1	712 ± 72	727 ± 75	710 ± 67	713 ± 57
Block 2	668 ± 77	679 ± 84	662 ± 76	664 ± 64
Block 3	650 ± 76	655 ± 85	648 ± 77	643 ± 62
Block 4	639 ± 74	639 ± 83	631 ± 71	626 ± 62

Data S1: Confidence judgments and explicit knowledge assessments

We examined whether participants' confidence ratings on the 2AFC task were related to their accuracy. Confidence was significantly higher for accurate than inaccurate 2AFC responses for context-dependent trials in both experiments (Unsignaled: $t(49) = 4.1$, $p < 0.001$; Signaled: $t(49) = 3.96$, $p < 0.001$) and on context-independent trials in Expt. 1 (Unsignaled; $t(37) = 2.6$, $p = 0.013$) but not Expt. 2 ($t(39) = 0.38$, $p = 0.7$) (Fig. S2A). Participants who responded entirely correctly or incorrectly were excluded from analysis; all participants had mixed accuracy on context-dependent trials. Overall, mean confidence ratings were around or below the midpoint of the scale.

On the Structure Knowledge Probe, two independent raters coded all responses with 91% agreement; discrepancies were resolved by deferring to the more senior grader. Explicit knowledge of the pair structure was identified in 34.0% of participants in Expt. 1 and 40.0% in Expt. 2.

Participants reported an average of 7.4 ± 4 in Expt. 1 and 7.9 ± 4 in Expt. 2 on the Pair Reconstruction Task (Fig. S2B). Group-level significance was calculated as the average number of correct context-independent pairs was greater than expected by chance over the simulations of all possible pair entry counts. Context-dependent pair entry performance was non-significant for both experiments (Unsignaled: mean = 2.06 pairs; $p = 0.11$; Signaled: mean = 2.08 pairs; $p = 0.11$; Fig. S2B). Moreover, only 36% of Expt. 1 participants and 34.7% of Expt. 2 participants (e.g., 17 out of 49 participants; one participant did not complete this portion of the experiment) reported the context-independent pair.

Methods S1: Model architecture comparison

In the main paper, we analyze a GRU model with training that was constrained to a single epoch, equivalent to the total sequence exposure of each human participant. Here, we justify that decision with comparison to two simpler models: a feedforward neural network (FFNN) and a vanilla recurrent neural network (RNN) that lacked gated recurrent units. One learning phase sequence and one set of 2AFC questions were generated for each model in the same way as for human participants (1,600 objects), and each epoch of training consisted of updating model weights to predict the next item in this sequence, followed by an assessment of 2AFC accuracy with frozen weights. For each model, we continued this process for a total of 50 epochs (i.e. 50 times the sequence exposure given to human participants). All models here used the default PyTorch weight initialization where weights are drawn from a uniform distribution bounded by plus or minus the inverse of the square root of the layer size, which was 0.08 for the hidden layer.

The simplest architecture, the FFNN, achieved an overall context-dependent accuracy of almost 75% (Fig. S3A). However, this performance was entirely driven by near-perfect accuracy on Context B, the most recently trained context, while accuracy on Context A remained near chance. This indicates that the FFNN retained knowledge only about the most recent associations, completely overwriting previously learned, conflicting ones—a hallmark of catastrophic interference.

RNNs improve on feedforward model capabilities by incorporating information from past hidden states with the current state, enabling them to process sequential input. However, the RNN showed no improvement in overall context-dependent accuracy compared to the FFNN (Fig. S3B). While Context A performance did increase over learning, this improvement came at the expense of Context B performance, suggesting the RNN is also prone to interference.

The RNN with GRUs, an advanced RNN variant, overcomes the limitations by using update and reset gates to manage long-term dependencies more effectively. Initially, GRU performance was comparable to the FFNN (Fig. S3C). However, with extended training (approximately 20 epochs; i.e., 20 times the exposure of human participants), the GRU achieved comparably high accuracy on both Context A and Context B. This performance likely stems from the GRU's architectural advantages. The update gate controls how much new input influences retained memory, allowing the model to ignore unreliable input (such as noisy between-pair transitions). The reset gate allows the selective clearing of irrelevant information in response to context changes, thereby avoiding interference from outdated associations. Given that the GRU model provides the best account for context-dependent learning in humans, we used the GRU model for all of the single-epoch modeling analyses reported in the main text.

Methods S2: GRU model performance with perceptual object representations

The main paper used one-hot vector representations for each object in the modeling analysis. This choice ensured that all objects were represented equally and orthogonally, such that any structure emerging in the hidden layer reflected purely learned associations rather than preexisting similarities among the inputs. Here, we present the same analysis using perceptual object representations that more closely approximate the visual experience of human participants in the task. Perceptual object representations were generated by inputting each object image (without the overlaid plus or minus symbol) into AlexNet (1) and then applying PCA to reduce the dimensionality to 11 dimensions, matching the number of input and output dimensions of the original model. GRU networks were trained on the same context-dependent sequential prediction task as in the main text, using cosine similarity as the loss function, and assignment of objects to specific pairs was randomized for each model in the same way as for human participants.

As shown in Fig. S4, the relationship between initialized weight variance and 2AFC accuracy retained the same non-monotonic profile observed in the models that used one-hot input coding (Fig. 4B). In addition, 2AFC performance was higher for all question subsets. This improvement is unsurprising: the use of AlexNet embeddings introduces a strong visual prior that allows the model to exploit shared perceptual features when making predictions, thereby obscuring the interpretability of how the hidden layer activity represents the task's temporal associative structure. For example, the model could leverage arbitrary similarities in dimensions such as shape and color to bias its 2AFC responses. In contrast, one-hot encodings constrain all non-active dimensions to zero, ensuring that any hidden layer structure arises exclusively from learning the task's associative regularities. Taken together, these results confirm that the core finding of optimal task performance emerging at moderate weight initialization variance holds regardless of input representation. We therefore focus analysis on models trained with one-hot object encodings because they provide a controlled representational space in which hidden layer structure reflects learning task's associative structure rather than preexisting perceptual relationships among stimuli.

Methods S3: GRU performance with a fully overlapping stimulus set (no context-specific objects)

The pair assignment across contexts for both models and human participants included one possible caveat to our claim of latent context-dependent learning: for one of the context-dependent pair types, the second (paired) item appeared in only one of the contexts while the first item occurred in both (visualized in bottom row of Fig. 1B). This structure still required humans and the models to update their prediction of the second item based on the inferred context (consistent with all other context-dependent pairs), but also meant that the context-specific second item could have been used as a context cue independent of recent sequence history (Expt. 1) and/or border color (Expt. 2). In other words, an encounter with a context-specific object could be an indicator that the state of the world has changed and thus that one's associative predictions should be updated. We note that our decision to include this pair type was motivated by our intention to collect fMRI data with this paradigm, which will allow us to assess changes in neural representational geometry when an object's associative identity remains constant across contexts, providing a baseline for evaluating relative changes in other pair conditions.

To evaluate whether the presence of context-specific objects influenced model learning, we ran neural network simulations in which such pairs were removed and replaced with context-dependent pairs for which both objects could occur in either context. These models were trained on the same task, but the context-dependent pairs were reconfigured to maintain the overall object set, with second-item assignments shuffled across contexts. Model input and output dimensions were therefore reduced to 10, corresponding to the 10 unique object encodings needed to instantiate this modified pair set. All other training parameters and analysis of 2AFC performance were identical to those in the main text.

As shown in Fig. S5, model performance across weight initializations closely mirrored the results of the main analysis (Fig. 4B), indicating that learning dynamics and context-dependent accuracy were unaffected by the presence or absence of the context-specific object. This indicates that such objects did not serve as reliable context cues for our models.

Methods S4: Determination of hidden layer size

The main paper analyzes a GRU model with 150 hidden layer units. To assess whether model capacity influenced learning performance, we trained GRU models with reduced hidden layer sizes of 50 and 100 units. As shown in Fig. S6, all models ultimately achieved comparable performance converging to approximately 90% accuracy. However, models with fewer hidden layer units exhibited slower learning trajectories, requiring more training to reach the same level of performance as the model with 150 units. These results suggest that while increasing the number of hidden units accelerates learning, overall task performance is largely independent of model size.

Supplemental References

1. A. Krizhevsky, I. Sutskever, G. E. Hinton, ImageNet Classification with Deep Convolutional Neural Networks in *Advances in Neural Information Processing Systems*, (Curran Associates, Inc., 2012).
